# RETRACTION: Predicted Factors of Surgical Site Infection in Glioblastoma Patients: A Meta‐Analysis

**DOI:** 10.1111/iwj.70570

**Published:** 2025-04-18

## Abstract

**RETRACTION:** GuZ. , WangQ. , ChenJ. , and ZhuY. , “Predicted Factors of Surgical Site Infection in Glioblastoma Patients: A Meta‐Analysis,” International Wound Journal 21, no. 3 (2024): e14504, 10.1111/iwj.14504.38044279 PMC10898386

The above article, published online on 03 December 2023, in Wiley Online Library (http://onlinelibrary.wiley.com/), has been retracted by agreement between the journal Editor in Chief, Professor Keith Harding; and John Wiley & Sons Ltd. Following an investigation by the publisher, all parties have concluded that this article was accepted solely on the basis of a compromised peer review process. In addition, the investigation found significant textual overlap between this article and another article by different authors [1]. The editors have therefore decided to retract the article. The authors did not respond to our notice regarding the retraction.
